# Site-directed mutagenesis of *Campylobacter concisus* respiratory genes provides insight into the pathogen’s growth requirements

**DOI:** 10.1038/s41598-018-32509-9

**Published:** 2018-09-21

**Authors:** Stéphane L. Benoit, Robert J. Maier

**Affiliations:** 1 Department of Microbiology, University of Georgia, Athens, 30602 Georgia; 2 Center for Metalloenzyme Studies, University of Georgia, Athens, 30602 Georgia

## Abstract

*Campylobacter concisus* is an emerging human pathogen found throughout the entire human oral-gastrointestinal tract. The ability of *C*. *concisus* to colonize diverse niches of the human body indicates the pathogen is metabolically versatile. *C*. *concisus* is able to grow under both anaerobic conditions and microaerophilic conditions. Hydrogen (H_2_) has been shown to enhance growth and may even be required. Analysis of several *C*. *concisus* genome sequences reveals the presence of two sets of genes encoding for distinct hydrogenases: a H_2_-uptake-type (“Hyd”) complex and a H_2_-evolving hydrogenase (“Hyf”). Whole cells hydrogenase assays indicate that the former (H_2_-uptake) activity is predominant in *C*. *concisus*, with activity among the highest we have found for pathogenic bacteria. Attempts to generate site-directed chromosomal mutants were partially successful, as we could disrupt *hyfB*, but not *hydB*, suggesting that H_2_-uptake, but not H_2_-evolving activity, is an essential respiratory pathway in *C*. *concisus*. Furthermore, the tetrathionate reductase *ttrA* gene was inactivated in various *C*. *concisus* genomospecies. Addition of tetrathionate to the medium resulted in a ten-fold increase in cell yield for the WT, while it had no effect on the *ttrA* mutant growth. To our knowledge, this is the first report of mutants in *C*. *concisus*.

## Introduction

*Campylobacter concisus* is a Gram-negative ε-proteobacterium that was first isolated by Tanner and coworkers in 1981 from human gingival crevices of a patient with gingivitis^1^. *C*. *concisus* is commonly found in the oral environment of healthy individuals^2^ although it is not considered to be a dominant oral species. It is frequently associated with periodontitis, gingivitis and other dental diseases^3^. A recent study also found elevated levels of *C*. *concisus* in the microbiome of potentially malignant oral leukoplakia^4^. The presence and spectrum of action of *C*. *concisus* are not strictly limited to the oral cavity though. Indeed, data collected within the last 20 years indicate that *C*. *concisus* can be found throughout the entire gastrointestinal tract (GIT), including (i) the esophagus: high levels of *C*. *concisus* were found in 57% of patients with Barrett’s esophagus (BE) syndrome (but none in the control subject), suggesting a link between presence of the bacterium and BE^5^; (ii) the gastric mucosa: *C*. *concisus* is highly active within the gastric fluid (an increase of 444% compared to the total microbiota), irrespective of pH^6^. Furthermore, *C*. *concisus* pathotypes are present at significant levels in patients with gastroenteritis^7^; (iii) the intestines, including the ileum, jejunum, cecum and rectum^3^. Besides, higher prevalence of *C*. *concisus* were found in children with Crohn’s disease, as well as in adults with inflammatory bowel disease (IBD)^8^. In addition, *C*. *concisus* has been shown to be associated with intestinal pathogenicity in immunocompromised patients^9^. While they are phenotypically indistinguishable, most *C*. *concisus* strains show high degree of genetic variability, irrespective of their preferred niche (oral or enteric) or the diseases they cause. Thus, *C*. *concisus* strains can be classified in two main genomospecies, based on various typing methods that include amplified fragment length polymorphisms (AFLP)^10,11^, 23 S rRNA PCR^11^ and multi-locus sequence typing (MLST)^12–14^. The latter method has proven to be also useful to discriminate *C*. *concisus* against other emerging *Campylobacter* species^15^.

The distribution of *C*. *concisus* throughout the entire GIT suggests a highly adaptable metabolism, as well as versatile respiratory pathways. Indeed, Tanner *et al*. first described the organism as “being able to grow under both microaerophilic (5% O_2_) or anaerobic conditions”, with “formate and H_2_ used as energy sources”^1^. Since then, *C*. *concisus* has been systematically described as a H_2_-requiring microorganism^16,17^, and the use of H_2_-enriched gas mixture, and more specifically H_2_-enriched microaerobic gas mixtures have become standard practice to grow *C*. *concisus*, highlighting the importance of H_2_ (and by extension, hydrogenases) in the pathogen’s metabolism. Results from Lee and coworkers indicated that H_2_ is required for *C*. *concisus* to grow under microaerobic conditions, as none of the 63 *C*. *concisus* strains tested in the study were able to grow on plates unless H_2_ was added^18^. The same study found that most *C*. *concisus* strains tested could grow under anaerobic conditions without H_2_, however presence of H_2_ in the gas mixture significantly enhanced the pathogen’s growth^18^. Thus, it appears that H_2_ is required for optimal growth of *C*. *concisus*, especially in presence of microaerobic O_2_ levels.

Based on analysis of multiple *C*. *concisus* genome sequences, the pathogen appears to possess two hydrogenase operons. One, annotated as “*hyd*”, encodes for a hydrogenase that shares high sequence homology with H_2_-uptake type hydrogenases found in other ε-proteobacteriae, such as *Helicobacter pylori*^19^, *Helicobacter hepaticus*^20^, *Campylobacter jejuni*^21^ or *Wolinella succinogenes*^22^; the second one, annotated as “*hyf*”, encodes for a putative H_2_-evolving type hydrogenase similar to Hyd-3 and Hyd-4 complexes found in *E*. *coli*^23^. Together, Hyd-3 and formate dehydrogenase H (FDH-H) form the formate hydrogenlyase (FHL) complex which disproportionates formate to H_2_ and CO_2_ under mixed acid fermentative conditions in *E*. *coli*^24^. Although the exact role of Hyd-4 remains elusive, its subunit composition and its homology to Hyd-3 suggest it can also form a FHL-like complex, called FHL-2^25^. Both FHL and FHL-2 are structurally related to the NADH dehydrogenase complex I of the respiratory chain^26^. In addition, *C*. *concisus* possesses a third operon (“*hyp*”), with genes encoding putative hydrogenase accessory proteins needed for maturation of both hydrogenases. The *hyp* operon is located on the same locus as the *hyd* operon.

In the present study, we aimed at generating mutants in genes belonging to each of the three aforementioned operons, *e*.*g*. *hyd*, *hyf* and *hyp*, a technical challenge since, to our knowledge, no *C*. *concisus* mutant has been reported yet. While attempts using conventional methods (electroporation or natural transformation) failed to deliver mutants, methylation treatment of the target DNA (using *C*. *concisus* cell-free extracts) prior to transformation proved successful. The construction and characterization of H_2_-evolving hydrogenase *hyfB* and tetrathionate reductase *ttrA* mutants show it is possible to inactivate genes in *C*. *concisus* by site-directed mutagenesis. Our results highlight the diversity of respiratory pathways in general and the importance of H_2_ metabolism in particular in this emerging pathogen.

## Results

### Analysis of *C*. *concisus* genome sequence reveals a versatile respiratory system that includes two hydrogenases

**Figure 1:**
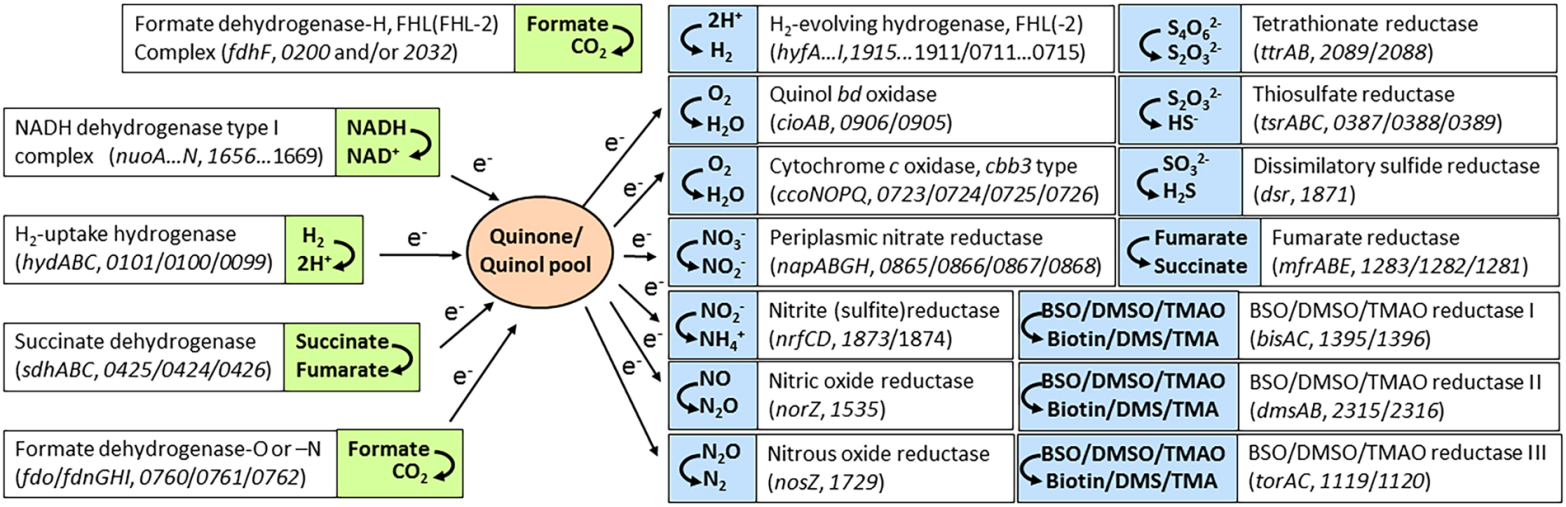
Putative respiratory pathways of *C*. *concisus*. Putative genes encoding for structural subunits of each respiratory enzyme complex are shown. Electron donors are shown in green and electron acceptors are shown in blue. Locus tag numbers and gene annotations refer to strain 13826 (BAA-1457), according to the JGI-IMG/M website (*img*.*jgi*.*doe*.*gov*). FHL: formate-hydrogenlyase complex. FHL-2: formate-hydrogenlyase 2 complex NADH: Nicotinamide Adenine Dinucleotide. BSO: biotin sulfoxide. DMSO: dimethyl sulfoxide. DMS: dimethyl sulfide. TMAO: trimethylamine N-oxide. TMA: trimethylamine.

Genome sequence analysis of *C*. *concisus* ATCC strains 13826 (also known as BAA-1457) and 51562^27^ revealed the presence of full sets of genes needed for aerobic (microaerophilic) as well as anaerobic respiration (Fig. 1 and data not shown). Indeed, based on its genome sequence, it appears the pathogen can use a variety of electrons donors such as succinate, formate, hydrogen, and NADH, while the list of putative electron acceptors includes oxygen, fumarate, nitrogen-containing compounds (nitrate, nitrite, nitric oxide, nitrous oxide, possibly trimethylamine N-oxide) and sulfur-containing compounds, including tetrathionate, thiosulfate, and possibly dimethyl sulfoxide (Fig. 1).

**Figure 2:**
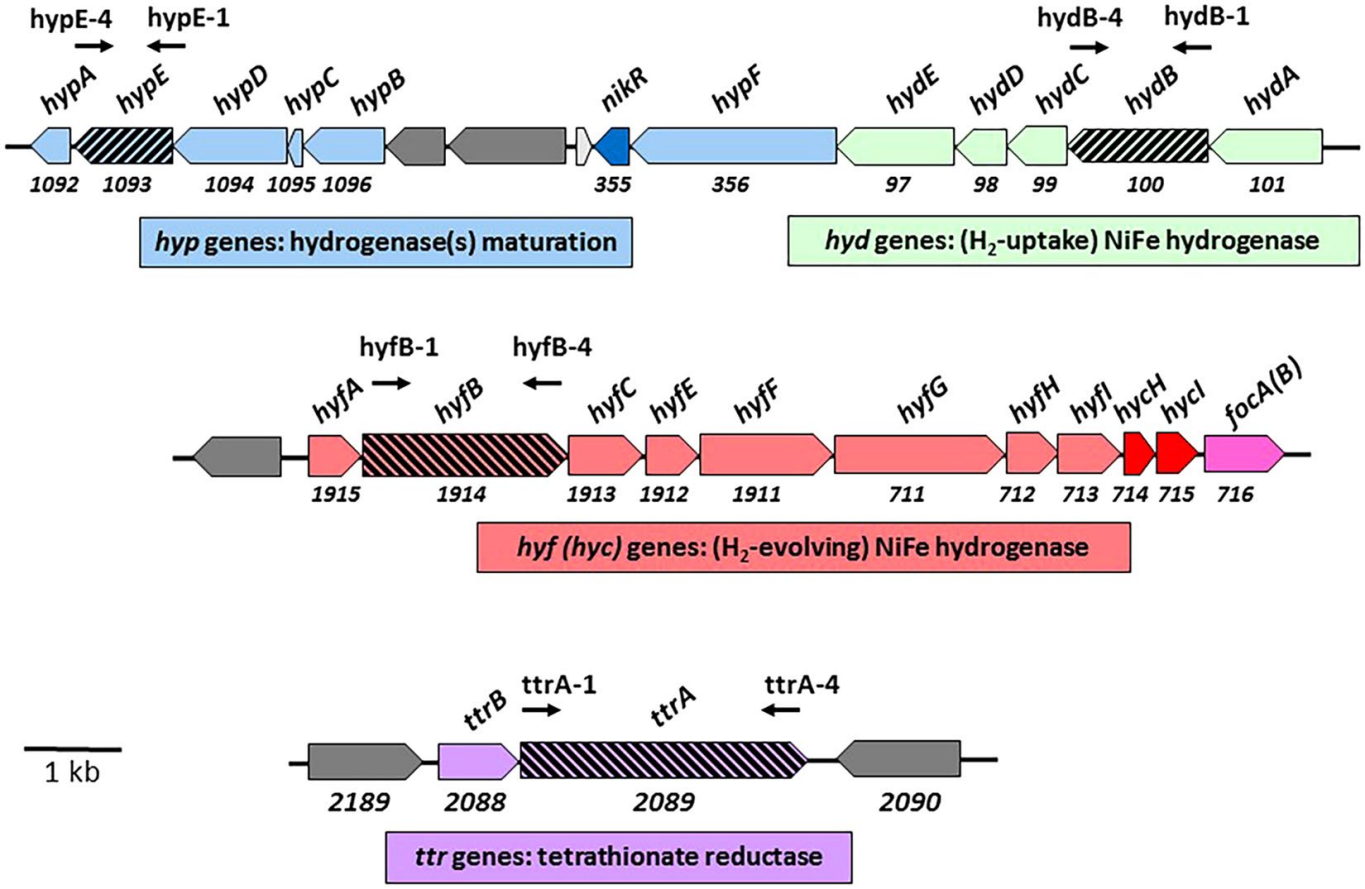
Genome location and organization of the *hyp*, *hyd*, *hyf* and *ttr* genes in *C*. *concisus* 13826 (BAA-1457). Genes annotations are according to the JGI-IMG/M website (*img*.*jgi*.*doe*.*gov*). Putative gene names are indicated above each gene. Numbers below each gene box indicate locus tag numbers. Genes targeted in this study (*hypE*, *hydB*, *hyfB*, *ttrA*) are indicated by dashed arrows. An approximate scale is shown bottom left.

Formate oxidation appears to be driven through two different formate dehydrogenases (FDH) in *C*. *concisus*: the first one has homology to FDH-N (or FDH-O), known to couple formate oxidation to nitrate reduction along with nitrate reductases^28^ and the second one has homology to FDH-H, usually found as part of the FHL complex^23^; both types of FDH are found in *Enterobacteriaceae*. Two sets of hydrogenase genes are present in *C*. *concisus* (Figs 1 and 2). *Hyd* genes encode for subunits of an H_2_-uptake hydrogenase, while *hyf*-annotated genes encode for a H_2_-evolving type 3 or 4 hydrogenase, the hydrogenase part of the FHL (or FHL-2) system in *E*. *coli*^23^. Indeed, the *C*. *concisus* “*hyf*” operon contains both *hyf* (*ABCEFGHI*) and *hyc* (*HI*) genes (Fig. 2) that are found in operons involved in Hyd-3 and Hyd-4 (H_2_-evolving) biosynthesis, respectively^23^. A study from Kovach *et al*. identified HyfI as being among the most immunoreactive proteins in *C*. *concisus*^29^. A 14-gene operon encoding for a full NADH dehydrogenase type I respiratory complex can be found. Two different sets of genes encoding for putative fumarate reductase (Frd)/succinate dehydrogenase (Sdh) enzyme complexes are present. The Cc13826_0424-0426 complex, herein annotated as SdhABC, is highly similar (80% identity) to *C*. *jejuni* Cj0408-410, previously shown to be a bifunctional Frd/Sdh enzyme^30^, while the Cc13826_1281-1283 complex (MfrABE) shares high homology with *C*. *jejuni* MfrABE (Cj437-0439), shown to have fumarate reductase activity only^30^. Regarding O_2_ respiration, *C*. *concisus* appears to have a branched respiratory chain, based on the presence of genes encoding for two terminal cytochrome oxidases, a *cbb3*-type and a *bd*-type quinol oxidase, respectively, similar to those found in *C*. *jejuni*^31^. Genes encoding for enzymes involved in respiration of various nitrogen compounds are present in all *C*. *concisus* strains: those include a periplasmic nitrate reductase (Nap), a nitrous oxide reductase (Nos), a nitric oxide reductase (Nor) and a putative periplasmic cytochrome *c* nitrite reductase (Nrf), although the latter is also hypothesized to be a polysulfite reductase^27,32^. In addition, *C*. *concisus* strains possess three sets of genes encoding for membrane bound, molybdenum (Mo)- or tungsten (W)-containing periplasmic enzymes that could be associated with either DMSO, TMAO or BSO respiration, as suggested by the concomitant presence of a Twin Arginine Translocation (TAT) signal peptide and a Mo/W-Bis-PGD binding motifs in their sequence. Finally, *C*. *concisus* has the capacity to respire sulfur-containing compounds, based on the presence of genes encoding for tetrathionate reductase (Ttr), thiosulfate reductase (Tsr), dissimilatory sulfide reductase (Dsr) and possibly sulfite reductase (Nrf), as discussed above (Fig. 1). The noticeable presence of high levels of hydrogen sulfide (H_2_S), one of the biochemical hallmarks of *C*. *concisus*^1^, confirms that the sulfur respiration pathway is operational. To coordinate these various pathways, *C*. *concisus* can rely on several putative transcriptional regulators, including putative CRP/FNR (13826_2145), NikR (13826_0355), Fur (13826_1795) and CsrA (13826_0062) regulatory proteins. Genome sequence analysis of the *C*. *concisus* GS2 strain 51562 confirmed the presence of all genes described above (data not shown), with the notable exception of the FDH-H gene (*fdhf*) homolog.

### H_2_ is required for optimal growth of *C*. *concisus* strain 13826 and 51562 both under anaerobic and microaerophilic conditions

**Figure 3:**
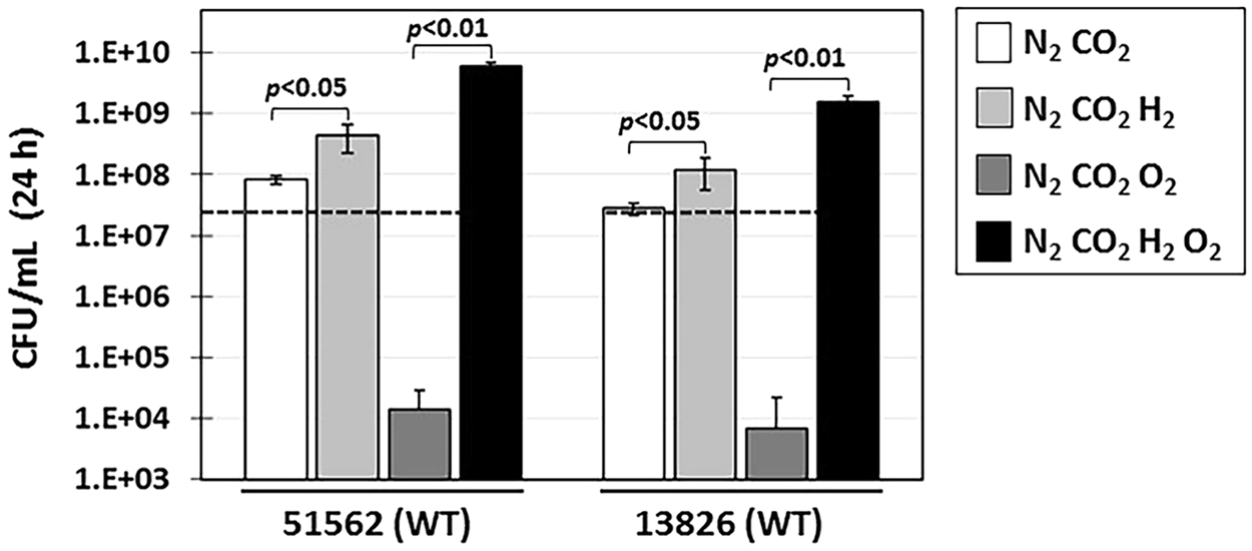
Effect of H_2_ on the anaerobic and microaerophilic growth of *C*. *concisus* WT strains 13826 and 51562. *C*. *concisus* WT strains 13826 and 51562 were grown for 24 h at 37 °C with vigorous shaking (200 rpm) in 165-mL sealed bottles containing 10 mL BHI broth supplemented with 10% fetal calf serum. Bottles were flushed with N_2_ for 15 min then CO_2_, O_2_ and/or H_2_ (5%, 5%, and 20% headspace partial pressure, respectively) were added in each bottle, as indicated on the right. After 24 h, growth yield was determined by measuring bacterial cell concentration, which is based on CFU counts after serial dilution in PBS, and is expressed as CFU/mL. The dashed line indicates the average inoculum for each strain, based on CFU counts. Columns and error bars represent mean and standard deviation, respectively, from three independent growth cultures. Statistically significant differences (Student’s *t*-test, two-tailed) are indicated above columns.

Previous results indicated that H_2_ plays a major role in *C*. *concisus* growth and we aimed at confirming these results with the two strains studied herein, using liquid cultures and well-controlled gas atmospheres. To determine the effect of H_2_ on the anaerobic or microaerophilic growth of strains 13826 and 51562, cells were inoculated in brain-heart infusion supplemented with fetal calf serum (BHI-FCS) liquid cultures in 165-mL bottles, with headspaces filled with four different gas atmospheric conditions (Fig. 3). After 24 h incubation at 37 °C under vigorous shaking, growth yield was determined (CFU/mL). Under anaerobic conditions there was modest growth for both *C*. *concisus* WT strains, however addition of H_2_ significantly enhanced cell yield (Fig. 3). The most dramatic effect of H_2_ was observed when cells were grown under microaerophilic conditions: neither strain grew under microaerophilic conditions without H_2_, while addition of H_2_ led to the highest growth yield observed herein (Fig. 3). Although formate also appears to be a potential electron donor due to the presence of FDH-N or –O genes (see Fig. 1), *C*. *concisus* cells did not grow in formate-supplemented medium under micraerobic conditions when H_2_ was absent (data not shown), suggesting that formate cannot substitute for H_2_ under these conditions. H_2_-enriched microaerophilic conditions are the most favorable growth conditions for *C*. *concisus*, as electrons generated by H_2_ oxidation flow along the respiratory chain with O_2_ as the final electron acceptor. Taken together, these results indicate that H_2_ is needed under anaerobic conditions to achieve optimal growth, while it is required under microaerophilic conditions, in agreement with results from a previous study^18^. These results demonstrate that *C*. *concisus* has (at least) one functional H_2_ uptake-type hydrogenase complex.

### Supplemental H_2_ induces protein synthesis and nutrient transport in *C*. *concisus*

To study the effects of supplemental H_2_ on protein synthesis in *C*. *concisus*, strain 51562 was grown on BA plates under H_2_-enriched microaerophilic atmosphere, or in liquid broth under the same four different gas mixture conditions as described above. Cells were harvested after 24 h, and the same amount (10 µg total protein) of cell-free extracts was loaded onto SDS-PAGE (Supplementary Fig. S1). Two bands, corresponding to H_2_-induced proteins with approximate molecular mass of 50 and 45 kDa, respectively, were excised from the gel and subjected to (MALDI-MS) peptide mass fingerprinting. The most abundant protein associated with the 45 kDa-protein band was identified as translation protein EF-Tu (ORF 51562_228, with a predicted mass of 43,628 Da). Interestingly, a previous study identified EF-Tu as one of the 37 most immunoreactive proteins in strain 13826^29^. Analysis of the 50 kDa-protein band revealed a major outer membrane protein from the OprD family (ORF 51562_1442, with a predicted mass of 46,399 Da) as the predominant protein. Other proteins associated with these two bands include other major outer membrane proteins, as well as hypothetical proteins (see Supplementary Table S3). Thus, these results suggest H_2_-derived energy can be used by *C*. *concisus* to bolster its protein synthesis and import more nutrients, with increased growth as the final outcome.

### *C*. *concisus* displays one of the highest H_2_-uptake hydrogenase activity recorded

In order to assess the H_2_-uptake activity in *C*. *concisus*, WT strains 13826 and 51562 cells were grown on plates under H_2_-enriched microaerophilic conditions and whole cell H_2_-uptake assays were carried out using an amperometric method, as previously described^19^. Hydrogenase activity levels ranged from approximately 115 to 200 nmoles of H_2_ used per min per 10^9^ cells (Table 1). Those H_2_-uptake activity levels are by far the highest recorded in our lab, between 3-to 60-fold higher than that previously measured (using the same amperometric method) for other pathogenic bacteria studied thus far (Table 1); those include *H*. *pylori*^20^, *H*. *hepaticus*^20^, *S*. *enterica* Typhimurium^33^ and *S*. *flexneri*^34^. Hydrogenase assays carried out in this study were done under aerobic conditions. Therefore, the elevated activity levels measured herein highlight the functionality of an extremely efficient respiratory electron transport chain in *C*. *concisus*.

**Table 1:** *C*. *concisus* H_2_-uptake hydrogenase activities of various pathogenic bacteria.

*Organism* (strain)	Whole cell H_2_-uptake activity*	Reference
*C*. *concisus* (13826)	113 ± 6	This study
*C*. *concisus* (51562)	199 ± 9	This study
*Helicobacter pylori* (26695)	33 ± 4	^20^
*Helicobacter pylori* (43504)	37 ± 2	^20^
*Helicobacter hepaticus* (51449)	3.2 ± 0.2	^20^
*Salmonella enterica* Typhimurium (14028 s)	12 ± 2	^33^
*Shigella flexneri*	68 ± 12	^34^

*H_2_-uptake activity is expressed as mean ± SD (nanomoles of H_2_ used per min per 10^9^ cells). All hydrogen uptake activities reported in this table were determined amperometrically with whole cells and O_2_ provided as the final electron acceptor. Results shown for *C*. *concisus* represent the mean ± SD of at least three independent assays.

### Construction and characterization of hydrogenase accessory *hyp* mutants

We sought to further understand the role played by H_2_ and hydrogenases in the pathogen’s metabolism by introducing mutations in putative hydrogenase genes. As stated above, it appears *C*. *concisus* possess three hydrogenase-related operons (Fig. 2): one operon contains *hyp* hydrogenase accessory genes probably needed for maturation of both hydrogenases; a second operon, annotated as *hyd*, is located on the same locus as the *hyp* operon and is predicted to encode for subunits of a H_2_-uptake type complex similar to that found in related ε -proteobacteria; the third operon, *hyf*, located elsewhere on the chromosome, possesses genes sharing significant homology with those of H_2_-evolving hydrogenases 3 (or 4). Since Hyp proteins are generally needed for maturation (and therefore activity) of all hydrogenase complexes, we aimed at abolishing both (H_2_-uptake and H_2_-evolving) hydrogenase activities at once by disrupting one of the *hyp* genes, *hypE* (Fig. 2). Attempts to disrupt the *hypE* gene using a *hypE::cat* PCR product (previously methylated with *C*. *concisus* cell-free extracts) proved unsuccessful. Additional attempts using an *E*. *coli* suicide plasmid containing *hypE::cat* were partially successful. Indeed, we were able to isolate chloramphenicol resistant clones, however PCR analysis revealed those were merodiploid mutants, with both a WT-like *hypE* and a (*hypE::cat*) mutant copy, following single cross-over insertion of plasmid DNA into the chromosome (data not shown). Furthermore, H_2_ uptake-type activity in the merodiploid mutants was similar to that of the WT (data not shown), suggesting the chromosomal copy of *hypE* was not disrupted. Similar merodiploid mutants have been described when essential genes, such at *nifU* or *tatC*^35,36^ were targeted in the related species *H*. *pylori*. While technical barriers cannot be ruled out, these results (or lack thereof) suggest *hypE* is an essential gene in *C*. *concisus*. One likely explanation is that it is required for the maturation of both hydrogenases, of which one (Hyd) seems to be also required, as suggested below.

### Construction and characterization of hydrogenase *hyd* and *hyf* mutants

Since the construction of *hyp* mutants proved to be a challenge, we aimed at constructing independent mutants in *hyd* and *hyf* operons by targeting *hydB* and *hyfB*, respectively (Fig. 2). The *hydB* gene encodes for the large subunit of the [NiFe] H_2_-uptake hydrogenase and the *hyfB* gene encodes for a multi-spanning transmembrane protein with significant homology (36% identity/56% similarity) to the subunit B of the H_2_-evolving hydrogenase-4 complex found in *E*. *coli*^25^. Independent PCR products containing *hydB::cat* or *hyfB::cat* were methylated, using *C*. *concisus* cell-free extracts specific for each strain, purified, and used to transform each respective parental strain (13826 or 51562). Chloramphenicol resistant cells were only obtained for *hyfB::cat* and with 13826 as parental strain. The concomitant insertion of the *cat* cassette and the partial deletion of the *hyfB* gene were confirmed by PCR (Fig. 3). Despite several attempts, we were unable to obtain *hydB::cat* mutants in either WT strain, even when plates were supplemented with 20 mM formate, suggesting that the H_2_-uptake hydrogenase complex is essential in *C*. *concisus*, while the H_2_-synthesis hydrogenase complex is not. H_2_ synthesis was determined in WT and *hyfB::cat* mutant, using (reduced) methyl viologen (MV) as the electron donor. WT strain (13826) displayed approximately 13.4 ± 3.1 µmoles of H_2_ produced per min per mg of protein, while MV-dependent hydrogenase activity in the *hyfB::cat* mutant was not detectable (<0.1 µmoles of H_2_/min/mg), confirming the H_2_-evolving hydrogenase pathway had been successfully inactivated in the *hyfB::cat* mutant. The growth of the *hyfB::cat* mutant was compared to that of the parental strain (13826) by using the same four different gas atmospheric conditions described above (anaerobic or microaerophilic, with or without supplemental H_2_). There was no significant difference in growth yield (CFU/mL after 24 h) between the WT and the *hyfB::cat*, as determined by cell counts (data not shown), suggesting the HyfB membrane protein does not play a major role in *C*. *concisus* under these conditions.

### Construction and characterization of *C*. *concisus* tetrathionate reductase mutants

**Figure 4:**
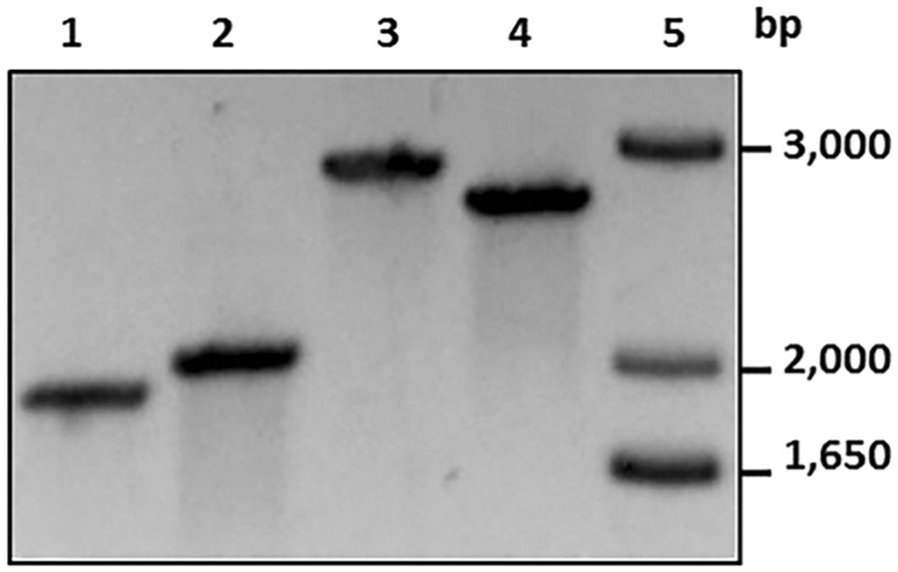
Agarose gel with PCR products used to verify cassette insertion. Lanes 1–4 contain PCR products amplified from genomic DNA of WT or mutant strains. Primers CchyfB-1 and CchyfB-4 were used to amplify *hyfB* from WT strain 13826 (lane 1, expected size: 1,925 bp) or *hyfB::cat* from 13826 Δ*hyfB::cat* mutant strain DNA (lane 2, expected size: 2,050 bp). Primers CcttrA-1 and CcttrA-4 were used to amplify *ttrA* from WT strain 51562 (lane 3, expected size: 2,900 bp) or *ttrA::cat* from 51562 Δ*ttrA::cat* mutant strain (lane 4, expected size: 2,675 bp). Lane 5 contains a DNA ladder, with sizes indicated on the right. The gel was stained with ethidium bromide. The picture has been digitally processed (black/white inverted).

The efficiency of the site-directed mutagenesis method was tested further on another putative respiratory gene, *ttrA* (Fig. 2). The *ttrA* gene encodes for the large subunit of the putative TtrAB tetrathionate reductase. Surprisingly, the *C*. *concisus* TtrA does not share homology with the bifunctional tetrathionate reductase/thiosulfate dehydrogenase TsdA found in the related species *C*. *jejuni*. Rather, its amino acid sequence resembles more that of the *Salmonella* Typhimurium (mono functional) tetrathionate reductase TtrA subunit (41% identity/56% similarity). In addition, *C*. *concisus* also possess genes encoding for a putative thiosulfate reductase (*tsrABC*, see Fig. 1). Following the same method used to generate *hyfB* mutants, we generated *ttrA::cat* mutants in both *C*. *concisus* WT genomospecies 13826 and 51562. The concomitant chromosomal insertion of the *cat* marker and partial deletion of *ttrA* was confirmed by PCR in both 51562 (Fig. 4) and 13826 (data not shown).

**Figure 5:**
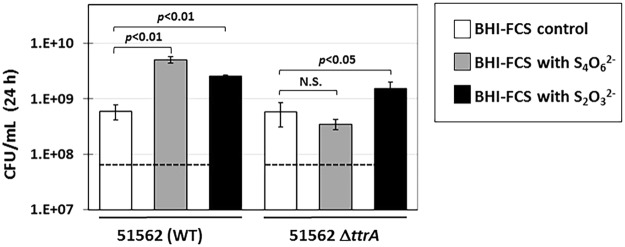
Effect of tetrathionate and thiosulfate on the anaerobic growth of *C*. *concisus* WT and Δ*ttrA* mutant strain. *C*. *concisus* 51562 WT and 51562 Δ*ttrA* mutant strains were grown in 10 mL BHI broth supplemented with 10% fetal calf serum and either 10 mM sodium tetrathionate (NaS_4_O_6_^2−^), or 15 mM sodium thiosulfate (NaS_2_O_3_^2−^), or none. Headspace contained 5% CO_2_, 20% H_2_ and 75% N_2_ (partial pressure). After 24 h at 37 °C under vigorous shaking, growth yield was determined in each bottle by determining cell concentration, which is based on CFU counts after serial dilution in PBS and is expressed as CFU/mL. Columns and error bars represent mean and standard deviation, respectively, from four independent growth cultures. The dashed line indicates the average inoculum for each strain, based on CFU counts. Statistically significant differences (Student’s *t*-test, two-tailed) are indicated above columns. N. S, not significant.

The effect of the *ttrA* mutation on *C*. *concisus* physiology was studied by growing cells from WT strain 51562 and its isogenic *ttrA::cat* mutant in liquid broth, under H_2_-enriched anaerobic conditions, with or without tetrathionate (S_4_O_6_^2−^) as terminal electron acceptor (Fig. 5). After 24 h incubation at 37 °C under vigorous shaking, growth yield was determined by counting colony forming units (CFU). Supplementation of the growth medium with 10 mM S_4_O_6_^2−^ resulted in almost 10-fold increase in cell yield for the WT strain (compared to the no S_4_O_6_^2−^ added condition), suggesting that S_4_O_6_^2−^ can be used as terminal acceptor under anaerobic conditions. In contrast, supplemental S_4_O_6_^2−^ had no effect on the growth yield of *ttrA* mutant cells (Fig. 5), indicating the targeted gene (*e*.*g*. *ttrA*) is indeed involved in S_4_O_6_^2−^ respiration. To determine whether the *ttrA* mutation has an effect on thiosulfate (S_2_O_3_^2−^) respiration as well, WT and *ttrA::cat* mutant cells were grown in the presence of 15 mM S_2_O_3_^2−^ (also in presence of H_2_). In this case, growth yield of all strains-WT and *ttrA::cat* alike-was significantly better compared to that of the control (no added terminal electron acceptor) and there was no difference between WT and mutant strains, indicating that both the WT and the *ttrA::cat* mutant can use S_2_O_3_^2−^ as terminal electron acceptor, under H_2_-enriched anaerobic conditions. In summary, the *ttrA* mutation prevented use of S_4_O_6_^2−^, while S_2_O_3_^2−^ metabolism was not affected.

## Discussion

To our knowledge, the present report is the first to describe the construction and characterization of mutants in the emerging pathogen *C*. *concisus*. Both strains chosen for this study, 51562 and 13826 (BAA-1457), are enteric strains that were originally isolated from feces of patients with gastroenteritis^1,37^. Both strains show great levels of genetic variability, to the extent that they belong to two distinct genomospecies (GS). Despite reports that BAA1457 is too atypical to be a reference strain^38^, we chose to include it in our study because its genome sequence is available and the strain belongs to the GS1 group^39^. Strain 51562 was also included in the present study, based on the facts that it is a sequenced strain and belongs to the GS2 group^27,39^.

Having a long-standing interest in H_2_ usage by pathogenic bacteria, such as *H*. *pylori*^19,20,40^, *H*. *hepaticus*^20,41^, S. *enterica* Typhimurium^33^ or *S*. *flexneri*^34^, we were particularly intrigued by reports on the H_2_ requirement in *C*. *concisus*. Indeed, previous studies suggested that not only H_2_ enhances *C*. *concisus* growth under anaerobic conditions, but also that H_2_ is actually required for *C*. *concisus* to grow in presence of microaerobic O_2_ concentrations^18^. Our first goal was to confirm the effect of H_2_ on *C*. *concisus* strains 13826 and 51562. Cells were grown in liquid cultures under N_2_-CO_2_ gas atmospheres, supplemented with defined volumes of H_2_, or/and O_2_, under vigorous shaking to increase gas diffusion throughout the growth medium. Our results confirmed that H_2_ is indeed required to achieve optimal growth under anaerobic conditions, and it is required to achieve growth under microaerophilic conditions, *e*.*g*. *C*. *concisus* cannot grow under microaerophilic conditions without H_2_. In fact, H_2_-supplemented microaerobic conditions appear to be the most favorable growth conditions (best yield) for *C*. *concisus*. This is in contrast with results from a previous study, which found that H_2_-supplemented anaerobic conditions are optimal for *C*. *concisus* growth^18^. The discrepancy between results from both studies could be attributable to several factors, including the use of different strains, as well as differences in the growth medium (solid or liquid), the gas-generating system and the quantity of H_2_ used. In agreement with the H_2_ requirement, both *C*. *concisus* strains 13826 and 51562 had extremely high H_2_-uptake hydrogenase activities, the highest recorded in our laboratory so far. Based on results obtained with both strains and the fact that genes encoding for the H_2_-uptake hydrogenase are present in all *C*. *concisus* genome sequences analyzed thus far (regardless of the GS they belong to), we hypothesize all members of the *C*. *concisus* species will have higher than usual H_2_-uptake hydrogenase activity. This will have to be experimentally verified though.

To get a better understanding of H_2_ metabolism in *C*. *concisus*, we aimed at inactivating hydrogenase maturation or synthesis genes using a classical site-directed mutagenesis approach. This, however, was anticipated to be a challenge since no mutant had been reported prior to the current study. A chloramphenicol resistance marker (*cat* gene) was chosen, based on the following: first, *C*. *concisus* has been shown to be Cm sensitive, with MIC of only 4 µg/mL reported in two independent studies^1,42^; second, the *cat* cassette was originally isolated from a related species, *Campylobacter coli*^43^; third, the cassette has been successfully used to generate numerous mutants (including hydrogenase mutants) in the related ε-proteobacteriaceae species *H*. *pylori* and *H*. *hepaticus*^40,41^; and fourth, the cassette has its own promoter and has been shown not to cause polar effects^36^. Therefore, the *cat* cassette appeared to be a suitable marker to disrupt genes in *C*. *concisus*. We aimed at inactivating both the H_2_-uptake and H_2_-synthesis hydrogenase pathways at the same time by targeting one of the *hyp* genes involved in hydrogenase maturation. Our first attempt to construct a *hypE::cat* mutant by natural transformation or electroporation was unsuccessful, suggesting that either the *hypE* gene was essential in *C*. *concisus*, or our transformation methods were not suitable for this microorganism, or both. Given that *C*. *concisus* belongs to the ε-proteobacterium group, a group whose members (*Helicobacters* or other *Campylobacters* for instance) are known to be naturally transformable, we hypothesized that transformation (DNA uptake) was not the reason our strategy was unsuccessful. Instead, the failure to introduce foreign DNA within *C*. *concisus* has probably more to do with its restriction/ modification system. Thus, we used a DNA methylation method originally developed to overcome the restriction barrier in *H*. *pylori*^44^. The method was successfully applied to *C*. *concisus*: first we were able to recombine *hypE::cat* along with a suicide plasmid into the chromosome. While this single cross-over recombination did not yield a *hypE* mutant *per se*, nevertheless it proved that both transformation and recombination into the *C*. *concisus* chromosome are possible, especially after proper methylation treatment. Using the same method and PCR products, we successfully inactivated two independent genes, *hyfB* and *ttrA*, encoding for a membrane component of the H_2_-evolving hydrogenase complex and the large subunit of tetrathionate reductase, respectively^27^.

**Figure 6:**
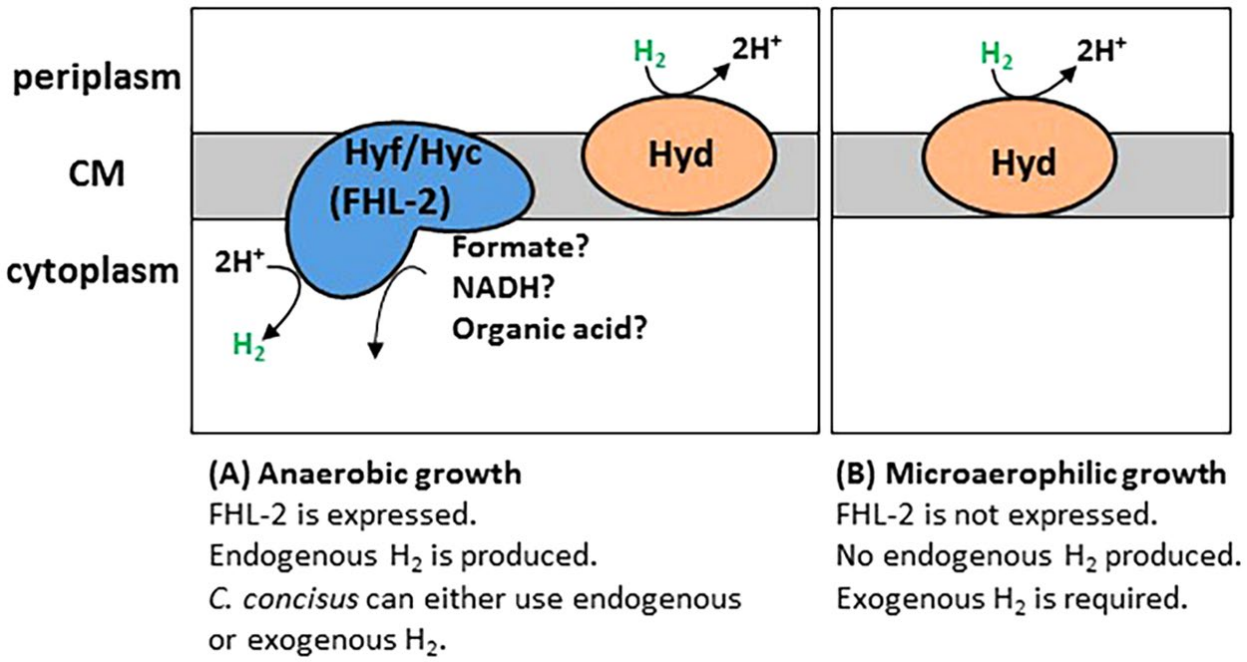
Model showing the redox-dependent expression of Hyd and Hyf hydrogenases complexes in *C*. *concisus*. Based on their protein subunit composition, both hydrogenase enzyme complexes are expected to be membrane-bound, however the presence of a TAT motif in the (HydA) sequence indicates the Hyd hydrogenase complex faces the periplasmic space, whereas the Hyf complex (as part of FHL) is supposed to be cytoplasmic. (**A**) Under anaerobic conditions both hydrogenase complexes are present, allowing the cells to use Hyf-produced H_2_ and grow without exogenous H_2_. (**B**) Under microaerophilic conditions, expression of FHL genes is inhibited and *C*. *concisus* only synthesizes Hyd. No growth of *C*. *concisus* under microaerophilic conditions is observed unless (host produced) exogenous H_2_ is available.

The use of molecular hydrogen as source of energy by bacteria, including human pathogenic bacteria, has been well documented (for a review, see^45^). However, in all pathogenic bacteria studied so far (*H*. *pylori*, *H*. *hepaticus*, *S*. *enterica* Typhimurium or *S*. *flexneri*), H_2_ is needed but it is not required, *e*.*g*. mutants devoid of H_2_-uptake hydrogenase activity are viable under laboratory conditions^34,40,41,46^. It seems this is not the case for *C*. *concisus*. The remarkable importance of H_2_ in the pathogen’s metabolism is highlighted by the fact that the H_2_-uptake hydrogenase appears to be essential for *C*. *concisus*, since we could neither inactivate *hypE*, a gene needed for maturation of hydrogenases in bacteria, including in *H*. *pylori*^47^, nor *hydB*, the gene encoding for the large subunit of the H_2_-uptake Hyd complex. The observation that *C*. *concisus* only requires exogenous (supplemented) H_2_ under microaerophilic conditions, but not under anaerobic conditions is puzzling, however it can be tentatively explained by the redox-dependent expression of the H_2_-evolving hydrogenase. Indeed, in *E*. *coli hyc* (Hyd-3) genes are only expressed under fermentative growth conditions *i*.*e*. in absence of all exogenous terminal electron acceptors, including O_2_^48^; likewise, *hyf* (Hyd-4) genes are also expressed under anaerobic conditions^49^. Applied to *C*. *concisus*, this means that *hyf* genes are likely to be expressed under anaerobic conditions, leading to endogenous production of H_2_ by the FHL or FHL-2 complex, as depicted in our proposed model (Fig. 6). Formate oxidation could be coupled to hydrogen production in *C*. *concisus*, as it is the case in *E*. *coli* with FHL^23^. Alternatively, other compounds (NADH or organic acids) could be oxidized instead of formate; indeed both the oxidized compound and the oxidizing enzyme (FDH-H counterpart) are still unknown with respect to the *E*. *coli* FHL-2 complex^23,26^. Regardless of whether formate or another electron donor plays a role in the *C*. *concisus* FHL-2 system, it appears *C*. *concisus* can produce H_2_, as shown in this study; this endogenous H_2_ could in turn be used by the Hyd hydrogenase, after diffusing through membranes. Based on this model one would expect the *hyf* mutant to have a lower growth yield compared to WT when cells are grown under anaerobic conditions, however there was no significant difference between strains, as both the WT and *hyf* mutant strains grew poorly, even in presence of formate. Thus, additional electron donors (*e*.*g*. NADH or organic acids, as discussed above) might be required to augment H_2_ synthesis and support anaerobic cell growth. In contrast, under aerobic or microaerophilic conditions, *hyc* or *hyf* genes are expected to be turned off, preventing cells from synthesizing H_2_^23^. Under these conditions, the only source of H_2_ *C*. *concisus* can rely on is exogenous H_2_ (Fig. 6). H_2_-enriched microaerophilic conditions are presumably the most favorable conditions for *C*. *concisus*, as electrons generated by H_2_ oxidation flow along the respiratory chain with O_2_ as the final electron acceptor (*C*. *concisus* possess both terminal cytochrome oxidases *cbb3* and *bd*). This was confirmed in the current study.

The fact that *C*. *concisus* can use H_2_ both under anaerobic and microaerophilic conditions likely explains why it can be found in various niches of the human body, including the oral cavity^2^. Despite the fact that this habitat is considered mostly anaerobic, *C*. *concisus* can probably rely on FHL-produced H_2_; in addition, exogenous H_2_ is also available, as suggested by several studies. For instance, colonic bacteria continuously produce H_2_ as part of their metabolism^50^; the gas is able to move into other tissues (including the lungs) through a combination of cross-epithelial diffusion^51^ and vascular-based transport^52^. As a consequence, approximately 14% of intestinal-produced H_2_ is predicted to be eventually excreted through the breath^53^. Furthermore, Kanazuru *et al*. found that the concentration of H_2_ in the oral cavity of non-expirating healthy volunteers was around 20–30 ppm, spiking to 120 ppm following glucose intake^54^. Most of this oral H_2_ was attributed to fermentation by *Klebsiella pneumoniae*. Such ranges correspond to millimolar H_2_ concentrations, which are high enough to be detected in the breath through a breath analyzer. While the affinity constant of *C*. *concisus* H_2_-uptake hydrogenase for the substrate (H_2_) is not yet known, most hydrogenases have a K_*m*_ in the micromolar range, therefore H_2_ levels in the oral cavity are likely not a limiting factor for *C*. *concisus* in the oral cavity; rather H_2_ is more likely to be found in excess.

Likewise, in the human gut, another natural niche for *C*. *concisus*, there is also abundant H_2_. Indeed, the colonic flora (predominantly composed of anaerobic bacteria) breakdown host-undigested carbohydrates, producing a variety of catabolites such as short chain fatty acids, lactate, CO_2_, formate and H_2_^50,55^. The latter can in turn be used by H_2_-uptake hydrogenase-containing bacteria, including pathogenic bacteria such as *C*. *concisus* and *S*. *enterica* Typhimurium. The role of hydrogenases in *S*. Typhimurium’s host colonization have been studied: Maier *et al*. showed that *S*.T. hydrogenase mutants are unable to colonize the colon of mice^56^. In addition, *S*. Typhimurium can respire S_4_O_6_^2−^, produced from host-driven S_2_O_3_^2−^ during inflammation^57^. The use of S_4_O_6_^2−^ as terminal electron acceptor confers *S*. Typhimurium a selective advantage over the competing microbiota that cannot respire S_4_O_6_^2– ^^57^. Thus, *S*. Typhimurium thrives under inflammatory conditions. Interestingly, *C*. *concisus* also possess a S_4_O_6_^2−^ reductase, that appears to be structurally closer to the *S*. Typhimurium enzyme than to the bifunctional (S_4_O_6_^2−^ reductase/ S_2_O_3_^2−^ oxidase) enzyme found in *C*. *jejuni*. In the present study, we were able to disrupt the *ttrA* gene encoding for the large subunit of S_4_O_6_^2−^ reductase in *C*. *concisus*. The phenotype associated with the mutation was as expected: addition of S_4_O_6_^2−^ enhanced growth in the WT, but not in the *ttrA* mutant. This suggests that the S_4_O_6_^2−^ reduction pathway is operational. Since *C*. *concisus*’s association with gut inflammatory diseases (such as ulcerative colitis and Crohn’s disease) is well documented^8,58^, it is very likely the pathogen can use host-produced S_4_O_6_^2−^ at its own advantage, similar to what has been described for *S*. Typhimurium. It is worth noting however that not all sequenced *C*. *concisus* strains possess tetrathionate reductase genes^27^.

Taken together, our results shed some light on *C*. *concisus*’s versatile respiratory system. Its H_2_-uptake and H_2_-synthesizing abilities, coupled to its capacity to respire nitrogen, sulfur and oxygen-containing compounds explain why *C*. *concisus* can successfully adapt to and colonize such diverse environmental niches of the human body. Finally, we showed that disrupting genes by site-directed mutagenesis in *C*. *concisus* is possible, and we hope this report will provide researchers with new genetic tools to study this emerging pathogen.

## Experimental Procedures

### Bacterial strains and plasmids

*E*. *coli* and *C*. *concisus* strains and plasmids used in this study are listed in Table S1. Genomic DNA from either *C*. *concisus* ATCC-51562 or *C*. *concisus* ATCC-BAA-1457 (13826) was used as template for all PCR amplifications. All plasmids and PCR products were sequenced at the Georgia Genomics Facility, University of Georgia, Athens, GA.

### Growth conditions

*Campylobacter concisus* was routinely grown on Brucella agar (Becton Dickinson, Sparks, MD) plates supplemented with 10% defibrinated sheep blood (Hemostat, Dixon, CA) (BA plates). Chloramphenicol (Cm, 8 µg/ml) was added as needed. Cells were grown at 37 °C, in sealed pouches filled with anaerobic mix, a commercial gas mixture containing 10% H_2_, 5% CO_2_ and 85% N_2_ (Airgas, Athens, GA). For liquid cultures, 165-ml sealed bottles were filled with 10 mL of Brain-Heart Infusion (BHI, Becton Dickinson) supplemented with 10% fetal calf serum (FCS, Gibco Thermo Fisher). To study the effect of H_2_ and O_2_ on *C*. *concisus* growth, bottles were first flushed with N_2_ for 15 min, then CO_2_ (5% headspace partial pressure, h.p.p.) was injected in every bottle, followed by H_2_ (20% h.p.p.) and/or O_2_ (5% h.p.p.), as indicated. To study the effect of tetrathionate or thiosulfate under anaerobic conditions, bottles were first sparged with N_2_ for 15 min, followed by anaerobic mix for 15 min. Additional H_2_ (10%) was added, then sodium tetrathionate (10 mM) or sodium thiosulfate (15 mM) were added as indicated. For all liquid growth experiments, the inoculum was prepared as follows: *C*. *concisus* cells grown on BA plates for less than 24 h were harvested, resuspended in BHI and standardized to the same OD_600_, before being inoculated (1:100) into 10 mL of BHI-FCS. The starting OD_600_ was between 0.03 to 0.04 (corresponding to 6.7 × 10^7^ to 9 × 10^7^ CFU/mL, respectively). The actual bacterial concentration at the time of inoculation was determined by plating serial dilutions and counting CFU. Cells were grown (triplicate or quadruplicate for each strain and condition) for 24 h at 37 °C under vigorous shaking (200 rpm). Growth yield (CFU/mL) was estimated as follows: samples from each bottle were serially diluted (up to 10^−7^) in PBS and 5 µL of each dilution was spotted in triplicate on BA plates. CFU were counted after 48 h of incubation under H_2_-enriched microaerophilic conditions. *E*. *coli* cells were grown aerobically in Luria-Bertani (LB) medium or plates at 37 °C, unless indicated otherwise. Ampicillin (100 µg/mL) and chloramphenicol (25 µg/mL) were added as needed.

### Identification of H_2_-induced proteins by MALDI-MS

*C*. *concisus* (strain 51562) was grown on BA plates under H_2_-enriched microaerophilic conditions or in BHI-FCS liquid broth, under four different gas atmospheres, as described above. After 24 h, cells were harvested, broken by sonication and spun down. Cell-free extracts were isolated and the protein concentration was determined with the BCA protein kit (Thermo Fisher Pierce, Rockford, IL, USA). Samples were processed using in-gel digestion with trypsin. MALDI was performed at the University of Georgia Proteomics and Mass Spectrometry (PAMS) Facility, on a a Bruker Autoflex using reflectron mode and 2,5-dihydrocybenzoic acid as matrix. Results were analyzed using the Mascot MS/MS ion search (Matrix Science, Boston, MA) and searches were performed on the National Center for Biotechnology Information (NCBI) non-redundant database (against genome sequences of *C*. *concisus* strains 51562 and 13826).

### Construction of *C*. *concisus* mutants

The construction of each mutant followed the same 3-step strategy: (1) generation of DNA constructs used for mutagenesis; (2) methylation of DNA constructs using *C*. *concisus* cell-free extracts and S-Adenosyl Methionine (SAM); (3) Transformation of *C*. *concisus* with the (purified) methylated DNA and selection on antibiotics-containing plates. In the first step, a splicing-by-overlap-extension (SOE) PCR method was used. Briefly, two DNA sequences ranging from 0.5 to 1-kb in size and flanking each target sequence (*hydB*, *hyfB*, *hypE* or *ttrA*, respectively) were amplified by PCR (iProof polymerase, Bio-Rad, Hercules, CA), using genomic DNA from strain 51562 and specific primers for each target (Table S1). Each set of two PCR products was then combined with a 740 bp-long *cat* (chloramphenicol resistance) cassette that has its own promoter^43^ and the final SOE PCR step yielded a product containing both flanking sequences with the *cat* cassette in the middle. In the second step, each tripartite PCR product was purified and methylated, following a modified method previously used to generate mutants in *H*. *pylori*^36,44,59,60^. Briefly, approximately 25 µg of DNA was incubated for 2 h at 37 °C with 150–250 µg of (cell-free extract) total protein from *C*. *concisus* (either strain 13826 or 51562, depending on final recipient strain) in presence of 0.4 mM of SAM (New England Biolabs, Ipswich, MA). After methylation, each PCR product was purified again (Qiaquick purification kit, Qiagen, Valencia, CA) and used to transform *C*. *concisus*; each strain was transformed by natural transformation or electroporation (BTX Transporator Plus, 2,500 V/pulse) with its corresponding strain-methylated DNA (1–5 µg). Transformed cells were first plated on BA plates and incubated (H_2_-enriched microaerophilic conditions) for 8–12 h before being transferred onto BA supplemented with 8 µg/mL Cm. Colonies appeared after 3 to 5 days. The concomitant deletion in the gene of interest (*hypE*, *hyfB* or *ttrA*) and the insertion of *cat* was confirmed by PCR, using genomic DNA from mutants as template and appropriate primers.

#### Construction of *hypE::cat* mutant

Primers CchypE-1 and CchypE-2 (Table S2) were designed to amplify a 500 bp-long DNA sequence corresponding to the first half of the *hypE* open reading frame (ORF) (13826_1093 or 51562_1332) as well as to incorporate the 5′ end of the *cat* marker. Primers CchypE-3 and CchypE-4 were designed to amplify a 520 bp-long sequence corresponding to the 3′ end of *cat* and the second half of the *hypE* ORF. The final SOE amplification step using CchypE-1 and CchypE-4 generated a 1,730 bp-long *hypE::cat* DNA sequence, with the *cat* cassette located in the middle of the *hypE* gene. The *hypE::cat* construct was either methylated and used to transform *C*. *concisus*, or it was cloned into plasmid pBluescript KS (pBS-KS). In this case, pBS-KS was digested with *Sma*I and ligated with the *hypE::cat* PCR product that had been previously blunt-ended with *T4* polymerase. The newly generated plasmid (plasmid pSB624, Table S1) was then methylated as described above prior to transformation.

#### Construction of *hydB::cat* mutant

Primers CchydB-1 and CchydB-2 (Table S2) were designed to amplify a 625 bp-long DNA sequence corresponding to the beginning of the *hydB* ORF (13826_0100 or 51562_1311) and the 5′ end of *cat*. Primers CchydB-3 and CchydB-4 were designed to amplify a 675 bp-long sequence corresponding to the 3′ end of *cat* and the 3′ end of the *hydB* ORF. The final SOE amplification step with both PCR products, the *cat* cassette and primers CchydB-1 and CchydB-4 generated a 2,000 bp-long *hydB::cat* DNA sequence, in which approximately 415 bp (of the 1,720 bp-long *hydB* ORF) is missing and replaced by *cat*.

#### Construction of hyfB::cat mutant

Primers CchyfB-1 and CchyfB-2 (Table S2) were designed to amplify a 700 bp-long DNA sequence corresponding to the beginning of the *hyfB* ORF (13826_1914 or 51562_0661) and the 5′ end of *cat*. Primers CchyfB-3 and CchyfB-4 were designed to amplify a 650 bp-long sequence corresponding to the 3′ end of *cat* and the 3′ end of the *hyfB* ORF. The final SOE amplification step with both PCR products, the *cat* cassette and primers CchyfB-1 and CchyfB-4 generated a 2,050 bp-long *hyfB::cat* DNA sequence, in which approximately 620 bp of the 1,925 bp-long *hyfB* ORF is missing and replaced by *cat*.

#### Construction of *ttrA*::cat mutant

Primers CcttrA1 and CcttrA2 (Table S2) were designed to amplify a 1,070 bp-long DNA sequence corresponding to the beginning of the *ttrA* ORF (13826_2089 or 51562_0690) and the 5′ end of *cat*. Primers CcttrA3 and CcttrA4 were designed to amplify a 920 bp-long sequence corresponding to the 3′ end of *cat* and the 3′ end of the *ttrA* ORF. The final SOE step with both PCR products, the *cat* cassette and primers CcttrA-1 and CcttrA-4 generated a 2680 bp-long *ttrA::cat* DNA sequence, in which approximately one third (970 bp out of 2,988 bp) of the core sequence of *ttrA* ORF is replaced by *cat*.

### Hydrogenase assays

#### Whole cells H_2_-uptake hydrogenase assays

H_2_-uptake was assayed using a previously described amperometric method^19^. Briefly, *C*. *concisus* cells from strain 13826 or 51562 were grown for 24 h on BA plates under H_2_-enriched microaerophilic conditions, harvested and resuspended in phosphate buffered saline (PBS). Cell density (OD_600_) was measured to evaluate cell concentration (1 unit of OD_600_ corresponds to approximately 2.25 × 10^9^ cells/mL, as determined in this study). A known volume of cells was injected into a 1.8 mL chamber containing H_2_-saturated PBS and H_2_ disappearance (H_2_ uptake by live *C*. *concisus* cells) was monitored as previously described^19^. Activities are reported as nmoles of H_2_ used per min per 10^9^ cells, and represent 3 to 4 independent measurements.

#### H_2_-evolving hydrogenase assays

H_2_-synthesis was measured by monitoring the oxidation of dithionite-reduced methyl viologen (MV, ε_604_ = 1.39 mM^−1^ cm^−1^) at 604 nm^61^. Briefly, *C*. *concisus* 13826 WT and 13826 Δ*hyfB* mutant strains were grown on BA plates under H_2_-enriched microaerophilic conditions. After 24 h, cells were harvested in N_2_-saturated HEPES-NaOH (50 mM) pH 7.5, broken by sonication and spun down for 5 min at 15,000 × *g*. Cell-free extracts were isolated and the protein concentration was determined using the BCA protein kit. MV (10 mM final concentration) was added to N_2_-saturated HEPES in 1.8 mL glass cuvettes closed with rubber stoppers. Freshly prepared sodium dithionite was injected to reduce MV and give a dark blue color with OD_604_ of approximately 1, and the reaction was initiated by adding 5 µl (20 to 40 µg) of cell-free extracts from either WT or Δ*hyfB* mutant cells. Activities are expressed as µmoles of H_2_ produced per min per mg of protein. Results represent means and standard deviations of three independent growth experiments, with assays done in triplicate.

### Genome sequence analysis

Gene and protein sequences of *C*. *concisus* strains 13826 (BAA-1457) and 51562 were obtained from the integrated microbial genomes (IMG) website of the Joint genome Institute (https://img.jgi.doe.gov). The following databases and prediction tools were also used: Genbank (www.ncbi.nlm.nih.gov), Uniprot (www.uniprot.org), and STRING (www.string-db.org).

## Electronic supplementary material

Supplementary data
