# Supplementary material for: Site-directed mutagenesis of Campylobacter concisus respiratory genes provides insight into the pathogen’s growth requirements
Source: Sci Rep. 2018 Sep 21;8:14203. doi: 10.1038/s41598-018-32509-9 (PMC6155014; doi:10.1038/s41598-018-32509-9)
Supplement: Supplementary file 1 — Supplementary data [file 41598_2018_32509_MOESM1_ESM.pdf]

<u>Supplementary data for</u>:

Site-directed mutagenesis of *Campylobacter concisus* respiratory genes provides insight into the pathogen's growth requirements

Stéphane L. Benoit[1,2]* and Robert J. Maier[1,2]

Department of Microbiology[1] and Center for Metalloenzyme Studies[2]

University of Georgia, Athens, Georgia, 30602

*To whom correspondence should be addressed:
Department of Microbiology,
804 Biological Sciences Bldg.,
University of Georgia
Athens, GA 30602,
United States of America
Tel.: 706-542-6875
Fax: 706-542-2674
E-mail: stefbens@uga.edu

Table S1. Strains and plasmids used in this study.

Strain or plasmid	Relevant characteristics	Source or reference
**Strain**		
E. coli		
TOP10	Cloning strain	Invitrogen
C. concisus		
13826	Parental strain. Genomospecies 1. Also known as BAA-1457.	ATCC
51562	Parental strain. Genomospecies 2.	ATCC (1)
**Plasmid**		
pBS-KS	pBluescript-KS cloning vector; Ap^r	Stratagene
pSB624	pBS-KS (*Sma*I) with *T4 pol* blunt-ended *C. concisus hypE::cat*; Ap^r Cm^r	This study
pUC20-cat	Source of *cat* cassette; Cm^r	(2)

References

1. Vandamme P, *et al.* (1989) Identification of EF group 22 campylobacters from gastroenteritis cases as *Campylobacter concisus*. *J Clin Microbiol* 27(8):1775-1781.
2. Wang Y & Taylor DE (1990) Chloramphenicol resistance in *Campylobacter coli:* nucleotide sequence, expression, and cloning vector construction. *Gene* 94(1):23-28.

Table S2. Primers used in this study.

Name	Sequence (5'-3')*	Usage
CchydB-1	TAGACCCTATAACACGTATCG	Construction of Δ*hydB::cat* mutant
CchydB-2	atccacttttcaatctatatcGCCATCATCTGAGCTGCTGTTC	Construction of Δ*hydB::cat* mutant
CchydB-3	cccagtttgtcgcactgataaGTTGATGGCGAGAGCATAGACG	Construction of Δ*hydB::cat* mutant
CchydB-4	TACTCAAATCATTTCCCTT	Construction of Δ*hydB::cat* mutant
CchyfB-1	CTTGTAAGTGCCGTCGTTAG	Construction of Δ*hyfB::cat* mutant
CchyfB-2	atccacttttcaatctatatcCGTTTGATGGTGCTGCTGGG	Construction of Δ*hyfB::cat* mutant
CchyfB-3	cccagtttgtcgcactgataaCTTTGCAGGTACTCCAAGAG	Construction of Δ*hyfB::cat* mutant
CchyfB-4	GCCCACAGCAAGTATGGCAC	Construction of Δ*hyfB::cat* mutant
CchypE-1	AGATAATGCTAAGCCACGGC	Construction of Δ*hypE::cat* mutant
CchypE-2	atccacttttcaatctatatcTTAAAATTTTAGCCCCTG	Construction of Δ*hypE::cat* mutant
CchypE-3	cccagtttgtcgcactgataaTCTCTGGAGATGTTGGCAGAC	Construction of Δ*hypE::cat* mutant
CchypE-4	TGCATTAGCAGATCCTTGGT	Construction of Δ*hypE::cat* mutant
CcttrA-1	CACGAGTGCTATGCTGGCAG	Construction of Δ*ttrA::cat* mutant
CcttrA-2	atccacttttcaatctatatAGTGTATCTCGCCTGCTAGC	Construction of Δ*ttrA::cat* mutant
CcttrA-3	cccagtttgtcgcactgataaCGGCGCGATCGACGTTGAGG	Construction of Δ*ttrA::cat* mutant
CcttrA-4	GTCTAGCGCAAGTGCCA CC	Construction of Δ*ttrA::cat* mutant

* Upper case letters indicate *C. concisus*-specific sequences and lower case letters indicate *cat* (Cm^r)-specific sequences. All primers were purchased from Integrated DNA Technology (IDT), Coralville, IA

Table S3: identification of H_2-induced proteins by MALDI-MS

A) Protein identification, lower band (approximate molecular mass: 45 kDa)

1. **Mixture 1** **Total score:** **274** **Expect:** 2.9e-023 **Matches:** 40
Components (only one family member shown for each component):
WP_021091496.1 **Mass:** 46371 **Score:** **204** **Expect:** 2.9e-016 **Matches:** 22
major outer membrane protein [Campylobacter concisus]
sp|A7ZCN0.1|EFTU_CAMC1 **Mass:** 43586 **Score:** **98** **Expect:** 1.3e-005 **Matches:** 12
RecName: Full=Elongation factor Tu; Short=EF-Tu
ORI07997.1 **Mass:** 39362 **Score:** 53 **Expect:** 0.38 **Matches:** 8
dihydroorotate dehydrogenase [Campylobacter concisus]

---

2. WP_021091496.1 **Mass:** 46371 **Score:** **204** **Expect:** 2.9e-016 **Matches:** 22
major outer membrane protein [Campylobacter concisus]
ERJ25524.1 **Mass:** 46371 **Score:** **204** **Expect:** 2.9e-016 **Matches:** 22
Major outer membrane protein [Campylobacter concisus ATCC 51562]
WP_084109357.1 **Mass:** 44922 **Score:** **81** **Expect:** 0.00057 **Matches:** 12
major outer membrane protein [Campylobacter concisus]
ORI02425.1 **Mass:** 44922 **Score:** **81** **Expect:** 0.00057 **Matches:** 12
hypothetical protein A3223_04625 [Campylobacter concisus]
WP_087580613.1 **Mass:** 44963 **Score:** **80** **Expect:** 0.00074 **Matches:** 12
major outer membrane protein [Campylobacter concisus]
OUT08258.1 **Mass:** 44963 **Score:** **80** **Expect:** 0.00074 **Matches:** 12
hypothetical protein B9N66_08195 [Campylobacter concisus]
ORI08998.1 **Mass:** 46461 **Score:** **75** **Expect:** 0.0024 **Matches:** 11
hypothetical protein A3835_01545 [Campylobacter concisus]
WP_087577689.1 **Mass:** 44939 **Score:** **72** **Expect:** 0.0048 **Matches:** 11
major outer membrane protein [Campylobacter concisus]
OUT13157.1 **Mass:** 44939 **Score:** **72** **Expect:** 0.0048 **Matches:** 11
hypothetical protein B9N63_07595 [Campylobacter concisus]
WP_054196905.1 **Mass:** 46478 **Score:** **67** **Expect:** 0.014 **Matches:** 11
major outer membrane protein [Campylobacter concisus]
ALF47951.1 **Mass:** 46478 **Score:** **67** **Expect:** 0.014 **Matches:** 11
major outer membrane protein [Campylobacter concisus]

---

3. WP_084042095.1 **Mass:** 45334 **Score:** **99** **Expect:** 8.5e-006 **Matches:** 14
major outer membrane protein [Campylobacter concisus]
ORI07533.1 **Mass:** 45334 **Score:** **99** **Expect:** 8.5e-006 **Matches:** 14
hypothetical protein A3854_05755 [Campylobacter concisus]

---

4. sp|A7ZCN0.1|EFTU_CAMC1 **Mass:** 43586 **Score:** **98** **Expect:** 1.3e-005 **Matches:** 12
RecName: Full=Elongation factor Tu; Short=EF-Tu
OUT16544.1 **Mass:** 43586 **Score:** **98** **Expect:** 1.3e-005 **Matches:** 12
elongation factor Tu [Campylobacter concisus]
OUT15130.1 **Mass:** 43600 **Score:** **98** **Expect:** 1.3e-005 **Matches:** 12
elongation factor Tu [Campylobacter concisus]
OUT14611.1 **Mass:** 43600 **Score:** **98** **Expect:** 1.3e-005 **Matches:** 12

elongation factor Tu [Campylobacter concisus]
OUT11572.1 **Mass:** 43600 **Score:** 98 **Expect:** 1.3e-005 **Matches:** 12
elongation factor Tu [Campylobacter concisus]
OUT06943.1 **Mass:** 43586 **Score:** 98 **Expect:** 1.3e-005 **Matches:** 12
elongation factor Tu [Campylobacter concisus]
OSQ25484.1 **Mass:** 43600 **Score:** 98 **Expect:** 1.3e-005 **Matches:** 12
elongation factor Tu [Campylobacter concisus]
ORI12453.1 **Mass:** 43600 **Score:** 98 **Expect:** 1.3e-005 **Matches:** 12
elongation factor Tu [Campylobacter concisus]
ORI08655.1 **Mass:** 43600 **Score:** 98 **Expect:** 1.3e-005 **Matches:** 12
elongation factor Tu [Campylobacter concisus]
ORI04319.1 **Mass:** 43600 **Score:** 98 **Expect:** 1.3e-005 **Matches:** 12
elongation factor Tu [Campylobacter concisus]
OJJ28386.1 **Mass:** 43600 **Score:** 98 **Expect:** 1.3e-005 **Matches:** 12
elongation factor Tu [Campylobacter concisus]
EAT97379.2 **Mass:** 43586 **Score:** 98 **Expect:** 1.3e-005 **Matches:** 12
translation elongation factor Tu [Campylobacter concisus 13826]
ALF47369.1 **Mass:** 43600 **Score:** 98 **Expect:** 1.3e-005 **Matches:** 12
translation elongation factor Tu [Campylobacter concisus]
WP_021090663.1 **Mass:** 43600 **Score:** 98 **Expect:** 1.3e-005 **Matches:** 12
elongation factor Tu [Campylobacter concisus]
ERJ31593.1 **Mass:** 43586 **Score:** 98 **Expect:** 1.3e-005 **Matches:** 12
Translation elongation factor Tu [Campylobacter concisus UNSW2]
ERJ27471.1 **Mass:** 43586 **Score:** 98 **Expect:** 1.3e-005 **Matches:** 12
Translation elongation factor Tu [Campylobacter concisus ATCC 51561]
ERJ27286.1 **Mass:** 43586 **Score:** 98 **Expect:** 1.3e-005 **Matches:** 12
Translation elongation factor Tu [Campylobacter concisus UNSWCS]
ERJ26269.1 **Mass:** 43600 **Score:** 98 **Expect:** 1.3e-005 **Matches:** 12
Translation elongation factor Tu [Campylobacter concisus ATCC 51562]
ERJ24367.1 **Mass:** 43586 **Score:** 98 **Expect:** 1.3e-005 **Matches:** 12
Translation elongation factor Tu [Campylobacter concisus UNSW1]
ERJ22755.1 **Mass:** 43586 **Score:** 98 **Expect:** 1.3e-005 **Matches:** 12
Translation elongation factor Tu [Campylobacter concisus UNSW3]
WP_002941132.1 **Mass:** 43586 **Score:** 98 **Expect:** 1.3e-005 **Matches:** 12
MULTISPECIES: elongation factor Tu [Campylobacter]
EIF06864.1 **Mass:** 43586 **Score:** 98 **Expect:** 1.3e-005 **Matches:** 12
Translation elongation factor Tu [Campylobacter concisus UNSWCD]
WP_087579859.1 **Mass:** 43555 **Score:** 85 **Expect:** 0.00022 **Matches:** 11
elongation factor Tu [Campylobacter concisus]
OUT10394.1 **Mass:** 43555 **Score:** 85 **Expect:** 0.00022 **Matches:** 11
elongation factor Tu [Campylobacter concisus]
WP_087585685.1 **Mass:** 43569 **Score:** 72 **Expect:** 0.0041 **Matches:** 10
elongation factor Tu [Campylobacter concisus]
OUT18952.1 **Mass:** 43569 **Score:** 72 **Expect:** 0.0041 **Matches:** 10
elongation factor Tu [Campylobacter concisus]

---

5. WP_087585699.1 **Mass:** 46439 **Score:** 65 **Expect:** 0.024 **Matches:** 10
major outer membrane protein [Campylobacter concisus]
OUT18970.1 **Mass:** 46439 **Score:** 65 **Expect:** 0.024 **Matches:** 10

hypothetical protein B9N61_02045 [Campylobacter concisus]
WP_021090830.1 **Mass:** 46605 **Score:** **65** **Expect:** 0.024 **Matches:** 10
major outer membrane protein [Campylobacter concisus]
ERJ26443.1 **Mass:** 46605 **Score:** **65** **Expect:** 0.024 **Matches:** 10
Major outer membrane protein [Campylobacter concisus ATCC 51562]
WP_072594872.1 **Mass:** 46608 **Score:** 54 **Expect:** 0.31 **Matches:** 9
major outer membrane protein [Campylobacter concisus]
OJJ28403.1 **Mass:** 46608 **Score:** 54 **Expect:** 0.31 **Matches:** 9
membrane protein [Campylobacter concisus]

---

6. WP_021085885.1 **Mass:** 45903 **Score:** **62** **Expect:** 0.049 **Matches:** 10
major outer membrane protein [Campylobacter concisus]
ERJ23351.1 **Mass:** 45903 **Score:** **62** **Expect:** 0.049 **Matches:** 10
Major outer membrane protein [Campylobacter concisus UNSW1]

---

7. ORI07997.1 **Mass:** 39362 **Score:** 53 **Expect:** 0.38 **Matches:** 8
dihydroorotate dehydrogenase [Campylobacter concisus]

Search parameters:

Type of search : Peptide Mass Fingerprint
Enzyme : Trypsin
Variable modifications : Oxidation (M)
Mass values : Monoisotopic
Protein Mass : Unrestricted
Peptide Mass Tolerance : ± 0.2 Da
Peptide Charge State : 1+
Max Missed Cleavages : 1
Number of queries : 56
Selected for scoring : 51

B) Protein identification, higher band (approximate molecular mass: 50 kDa)

1. WP_021090830.1 **Mass:** 46605 **Score:** **211** **Expect:** 5.8e-017 **Matches:** 23
major outer membrane protein [Campylobacter concisus]
ERJ26443.1 **Mass:** 46605 **Score:** **211** **Expect:** 5.8e-017 **Matches:** 23
Major outer membrane protein [Campylobacter concisus ATCC 51562]
WP_087585699.1 **Mass:** 46439 **Score:** **142** **Expect:** 4.6e-010 **Matches:** 17
major outer membrane protein [Campylobacter concisus]
OUT18970.1 **Mass:** 46439 **Score:** **142** **Expect:** 4.6e-010 **Matches:** 17
hypothetical protein B9N61_02045 [Campylobacter concisus]
ORI08672.1 **Mass:** 45867 **Score:** **105** **Expect:** 2.3e-006 **Matches:** 14
hypothetical protein A3835_03910 [Campylobacter concisus]
WP_054196414.1 **Mass:** 45825 **Score:** **88** **Expect:** 0.00011 **Matches:** 13
major outer membrane protein [Campylobacter concisus]
ALF47387.1 **Mass:** 45825 **Score:** **88** **Expect:** 0.00011 **Matches:** 13
major outer membrane protein [Campylobacter concisus]
WP_021085498.1 **Mass:** 46417 **Score:** 46 **Expect:** 1.8 **Matches:** 8
major outer membrane protein [Campylobacter concisus]
ERJ24443.1 **Mass:** 46417 **Score:** 46 **Expect:** 1.8 **Matches:** 8
Major outer membrane protein [Campylobacter concisus UNSW1]

2. WP_072594872.1 **Mass:** 46608 **Score:** **128** **Expect:** 1.2e-008 **Matches:** 18
major outer membrane protein [Campylobacter concisus]
OJJ28403.1 **Mass:** 46608 **Score:** **128** **Expect:** 1.2e-008 **Matches:** 18
membrane protein [Campylobacter concisus]

3. WP_087584277.1 **Mass:** 45307 **Score:** **94** **Expect:** 3.2e-005 **Matches:** 13
major outer membrane protein [Campylobacter concisus]
OUT11858.1 **Mass:** 45307 **Score:** **94** **Expect:** 3.2e-005 **Matches:** 13
hypothetical protein B9N62_02770 [Campylobacter concisus]

4. WP_021088086.1 **Mass:** 49448 **Score:** 54 **Expect:** 0.29 **Matches:** 9
3-deoxy-7-phosphoheptulonate synthase class II [Campylobacter concisus]

ERJ27442.1 **Mass:** 49448 **Score:** 54 **Expect:** 0.29 **Matches:** 9

2-keto-3-deoxy-D-arabino-heptulosonate-7-phosphate synthase II [Campylobacter concisus UNSWCS]
WP_054196693.1 **Mass:** 49506 **Score:** 54 **Expect:** 0.3 **Matches:** 9
3-deoxy-7-phosphoheptulonate synthase class II [Campylobacter concisus]

ALF47701.1 **Mass:** 49506 **Score:** 54 **Expect:** 0.3 **Matches:** 9

2-dehydro-3-deoxyphosphoheptonate aldolase (DAHP synthetase, class II) [Campylobacter concisus]

Search parameters:

Type of search : Peptide Mass Fingerprint
Enzyme : Trypsin
Variable modifications : Oxidation (M)
Mass values : Monoisotopic
Protein Mass : Unrestricted
Peptide Mass Tolerance : ± 0.2 Da
Peptide Charge State : 1+
Max Missed Cleavages : 1
Number of queries : 66
Selected for scoring : 48

Supplementary Fig. S1. Benoit and Maier, 2018

SDS-15% PAGE gel with proteins (cell-free extracts) from *C. concisus* 51562 grown under various gas conditions

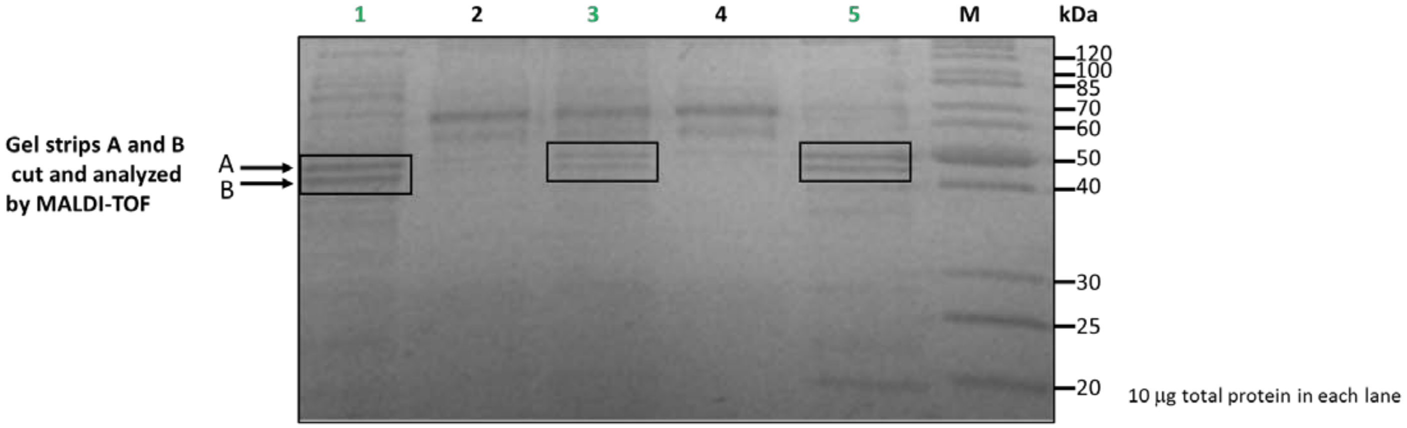

Lane	Growth Medium	Starting Gas concentration (%) *				Growth
		H_2	O_2	CO_2	N_2	
1	Solid (BA plate)	10	0-2	5	85	Good (standard growth condition)
2	Liquid (BHI-FCS)	0	0	5	95	OD_{600} (24h)=0.22
3	Liquid (BHI-FCS)	10	0	5	85	OD_{600} (24h)=0.32
4	Liquid (BHI-FCS)	0	5	5	90	OD_{600} (24h)=0.11
5	Liquid (BHI-FCS)	10	5	5	80	OD_{600} (24h)=0.89

* Cells grown on solid plates were incubated in a bag filled with 10% H_2, 5% CO_2 and 85% N_2, however residual O_2 in the pouch is a possibility.
